# Association of age, hormonal, and lifestyle factors with the Leydig cell biomarker INSL3 in aging men from the European Male Aging Study cohort

**DOI:** 10.1111/andr.13220

**Published:** 2022-07-11

**Authors:** Ravinder Anand‐Ivell, Kee Heng, Katie Severn, Leen Antonio, Gyorgy Bartfai, Felipe F. Casanueva, Ilpo T. Huhtaniemi, Aleksander Giwercman, Mario Maggi, Terence W. O'Neill, Margus Punab, Giulia Rastrelli, Jolanta Slowikowska‐Hilczer, Jos Tournoy, Dirk Vanderschueren, Frederick C. W. Wu, Richard Ivell

**Affiliations:** ^1^ School of Biosciences University of Nottingham, University Park Nottingham UK; ^2^ School of Mathematics University of Nottingham, University Park Nottingham UK; ^3^ Department of Chronic Diseases and Metabolism Laboratory of Clinical and Experimental Endocrinology KU Leuven Belgium; ^4^ Department of Endocrinology University Hospitals Leuven Leuven Belgium; ^5^ Department of Obstetrics, Gynaecology and Andrology Albert Szent‐Gyorgy Medical University Szeged Hungary; ^6^ Department of Medicine Santiago de Compostela University, Complejo Hospitalario Universitario de Santiago (CHUS), CIBER de Fisiopatología Obesidad y Nutricion (CB06/03), Instituto Salud Carlos III Santiago de Compostela Spain; ^7^ Institute of Reproductive and Developmental, Department of Metabolism, Digestion and Reproduction Imperial College London London UK; ^8^ Department of Translational Medicine Lund University Malmö Sweden; ^9^ Endocrinology Unit, “Mario Serio” Department of Experimental and Clinical Biomedical Sciences University of Florence Florence Italy; ^10^ Centre for Epidemiology Versus Arthritis The University of Manchester & NIHR Manchester Biomedical Research Centre, Manchester University NHS Foundation Trust Manchester UK; ^11^ Andrology Unit United Laboratories of Tartu University Clinics Tartu Estonia; ^12^ Department of Andrology and Reproductive Endocrinology Medical University of Łódź Łódź Poland; ^13^ Department of Geriatrics University Hospitals Leuven Leuven Belgium; ^14^ Department of Endocrinology Manchester University NHS Foundation Trust Manchester UK

**Keywords:** aging male, HPG axis, hypogonadism, INSL3, Leydig cell, testosterone

## Abstract

**Background:**

Aging in men is accompanied by a broad range of symptoms, including sexual dysfunction, cognitive and musculoskeletal decline, obesity, type 2 diabetes, cardiovascular disease and hypertension, organ degeneration/failure, and increasing neoplasia, some of which are associated with declining levels of Leydig cell‐produced testosterone. High natural biological variance, together with multiple factors that can modulate circulating testosterone concentration, may influence its interpretation and clinical implications. Insulin‐like peptide 3 is a biomarker of Leydig cell function that might provide complementary information on testicular health and its downstream outcomes.

**Objectives:**

To characterize insulin‐like peptide 3 as a biomarker to assess gonadal status in aging men.

**Methods and materials:**

A large European multicenter (European Male Aging Study) cohort of community‐dwelling men was analyzed to determine how insulin‐like peptide 3 relates to a range of hormonal, anthropometric, and lifestyle parameters.

**Results and discussion:**

Insulin‐like peptide 3 declines cross‐sectionally and longitudinally within individuals at approximately 15% per decade from age 40 years, unlike testosterone (1.9% per decade), which is partly compensated by increasing pituitary luteinizing hormone production. Importantly, lower insulin‐like peptide 3 in younger men appears to persist with aging. Multiple regression analysis shows that, unlike testosterone, insulin‐like peptide 3 is negatively dependent on luteinizing hormone and sex hormone‐binding globulin and positively dependent on follicle‐stimulating hormone, suggesting a different mechanism of gonadotropic regulation. Circulating insulin‐like peptide 3 is negatively associated with increased body mass index or waist circumference and with smoking, and unlike testosterone, it is not affected by weight loss in obese individuals. Geographic variation in mean insulin‐like peptide 3 within Europe appears to be largely explained by differences in these parameters. The results allowed the establishment of a European‐wide reference range for insulin‐like peptide 3 (95% confidence interval) adjusted for increasing age.

**Conclusion:**

Insulin‐like peptide 3 is a constitutive biomarker of Leydig cell functional capacity and is a robust, reliably measurable peptide not subject to gonadotropin‐dependent short‐term regulation and within‐individual variation in testosterone.

## 1 INTRODUCTION

Aging in adult men is a continuous process, largely correlating with the concentrations of circulating hormones that originate in or are dependent upon the testes. The best known of these hormones is testosterone (T), whose reduced concentrations have been linked to sexual dysfunction, obesity, metabolic syndrome, cardiovascular disease, cognitive decline, and physical frailty. However, there is considerable variability in the concentration of T, its biologically relevant proportion, and the responsiveness of various organ systems. Consequently, only clearly reduced levels of T, together with significantly increased symptoms of hypogonadism, are considered sufficient to constitute a clinical hypoandrogenemia (late‐onset hypogonadism [LOH] or more appropriately functional hypogonadism) warranting treatment.^1^

LOH, thus defined, represents only 2.1% of the community‐dwelling adult male population.^2^ How much circulating T is required for optimal health is unclear and must be seen in the context of lifestyle factors as well as morbidity. This is due in part to its bioavailability, because less than 2%, on average, of total T is not protein‐bound and thus available for interacting with specific androgen receptors (so‐called free testosterone [FT]). This is a parameter that in many conditions better correlates with disease incidence. Secondly, T varies across the day, and from day to day, and its synthesis by the testes is acutely feedback regulated by the hypothalamo‐pituitary‐gonadal (HPG) axis, which can compensate for declining T production by increasing the output of the gonadotropin luteinizing hormone (LH) as men age.

Although anatomical evidence indicates some loss of mature Leydig cells with age,^3^, ^4^ reduced T can also be because of declining hypothalamic or pituitary control, to reduced testicular sensitivity to LH, or to a deficiency of the testes to make T.^5^, ^6^ The last of these can be assessed from the ratio of circulating T to LH concentrations, a parameter also referred to as Leydig cell functional capacity, which markedly and progressively declines with age in men, beginning already at about age 30 years; like its component parameters, it is subject to high within‐individual variance. A major diagnostic problem is that low T and hypogonadal symptoms do not always match each other.^2^ Most men with low T have no symptoms, and those with hypogonadal symptoms often have normal T levels; only monitoring T may therefore not provide the most accurate diagnosis, and the applicability of other parameters, such as insulin‐like peptide 3 (INSL3), should be tested to improve diagnostic accuracy.

If we could better understand the multiple interrelationships impacting the age‐related decline in circulating T and its relationship to increasing age‐dependent morbidity, then appropriate intervention at an earlier age and applied to a larger proportion of men might be able to redress the functional decline with aging and its consequent socioeconomic as well as health burden. A specific biomarker of Leydig cell functional status, in addition to T, that is independent of other (extra‐testicular) modifying factors could contribute to a more objective assessment of the age‐related decline in male gonadal function.

INSL3 is a peptide hormone secreted into the systemic circulation by the steroidogenic Leydig cells of the testes.^7^ Importantly, and unlike T, INSL3 appears to be secreted in an acutely constitutive manner, simply reflecting the number and extant differentiation status of Leydig cells; it is independent of acute regulation by gonadotrophic hormones of the HPG axis.^7^, ^8^ Whereas the best‐known function of INSL3 is the regulation of testicular descent in the fetus,^9^ in the adult, it may act as a germ cell survival factor.^10^ It is also associated with the maintenance of bone strength; genetic defects for either INSL3 or its cognate receptor RXFP2 in mice and humans are linked to osteopenia and osteoporosis.^11^ INSL3 has additionally been suggested to ameliorate liver fibrosis^12^ as well as deteriorating kidney function.^13^

Following an initial cross‐sectional study of community‐living men in Australia,^14^, ^15^ the present investigation was aimed at developing a more detailed understanding of the relationship between circulating INSL3 and hormonal/lifestyle factors in a larger multicenter European population of community‐dwelling aging men. This population study takes advantage of the extensive phenotyping carried out in the EMAS cohort and additionally profits from the longitudinal nature of this study in addition to the cross‐sectional data.^16^ The results describe the cross‐sectional and longitudinal characteristics of INSL3 in the normal aging male population, in comparison with T, and reinforce its role as an independent biomarker of Leydig cell functional capacity.

## 2 MATERIALS AND METHODS

### 2.1 Subjects, anthropometric data

The first phase of the EMAS study recruited 3369 community‐dwelling men aged 40–79 years between 2003 and 2005, approximately equally distributed between eight European centers in northern, western, eastern, and southern Europe.^17^ In addition to basic anthropometric and hormonal parameters, subjects were also presented with postal and interviewer‐assisted questionnaires asking about the prevalence of disease, lifestyle factors, and physical and cognitive performance.^17^ Following the first phase, the men were recalled on average 4.3 years (range 3.0–5.7 years) later in 2008–2010 for the second, follow‐up phase,^16^ when essentially similar parameters were repeated. Altogether, for the second phase, 2736 men were assessed (81.2% retention); 106 men (3.2%) were considered too frail, 193 had died (5.7%), and 334 (9.9%) were lost to follow‐up. Ethical approval was obtained in accordance with local institutional requirements in each center.

For the present study, 2283 serum samples (83.4%) from the second phase were available for the measurement of INSL3, approximately equally distributed between all eight centers. From the first phase, only 1252 serum samples (37.2%) from four centers were available. Consequently, most analyses presented here represent the EMAS second phase, and samples from the first phase are only used for longitudinal comparison. Descriptive statistics for the parameters used for the second‐phase subjects analyzed here are given in Table 1 and do not differ from those presented previously^16^ for the entire cohort. Body weight was measured to the nearest 0.1 kg, and height to the nearest 1 mm. Body mass index (BMI) was calculated as kg/m^2^. Waist circumference (WC; cm) was measured three times for each subject, and the average was taken.

**TABLE 1:** Descriptive statistics of key parameters measured for phase 2 of the European Male Aging Study (EMAS) cohort by center

	Total	Leuven	Manchester	Santiago	Florence	Szeged	Malmo	Lodz	Tartu
Age (years)	63.0 ± 10.5 (2325)	62.9 ± 10.3 (245)	63.7 ± 10.8 (281)	62.9 ± 10.8 (292)	62.4 ± 10.2 (325)	62.6 ± 10.7 (346)	63.3 ± 10.6 (271)	63.8 ± 10.2 (262)	62.7 ± 10.7 (303)
INSL3 (ng/ml)	0.99 ± 0.50 (2283)	1.22 ± 0.55 (245)	1.10 ± 0.61 (281)	0.99 ± 0.48 (288)	0.99 ± 0.46 (325)	0.98 ± 0.47 (328)	0.96 ± 0.48 (263)	0.87 ± 0.45 (260)	0.81 ± 0.36 (293)
T (nmol/L)	16.38 ± 6.05 (2325)	17.91 ± 6.43 (245)	16.63 ± 5.63 (281)	16.39 ± 5.54 (292)	15.76 ± 5.22 (325)	15.39 ± 5.88 (346)	15.93 ± 6.31 (271)	16.89 ± 6.48 (262)	16.66 ± 6.66 (303)
cFT (pmol/L)	284.2 ± 90.2 (2283)	297.5 ± 90.5 (245)	292.9 ± 89.0 (281)	299.6 ± 85.8 (292)	280.2 ± 77.7 (325)	257.5 ± 82.1 (346)	298.0 ± 101.2 (230)	282.3 ± 107.5 (262)	276.0 ± 83.9 (302)
SHBG (nmol/L)	44.44 ± 20.10 (2283)	46.29 ± 21.36 (245)	41.91 ± 16.73 (281)	42.13 ± 18.39 (292)	43.36 ± 18.14 (325)	44.82 ± 21.78 (346)	42.62 ± 17.83 (230)	46.50 ± 20.59 (262)	47.84 ± 23.79 (302)
LH (IU/L)	6.29 ± 4.74 (2282)	5.85 ± 5.01 (244)	6.06 ± 5.03 (281)	6.17 ± 4.59 (292)	6.39 ± 4.63 (325)	6.56 ± 4.81 (346)	6.36 ± 4.79 (230)	6.46 ± 4.32 (262)	6.36 ± 4.71 (302)
FSH (IU/L)	8.51 ± 9.33 (2242)	7.80 ± 7.83 (244)	7.83 ± 9.83 (280)	8.07 ± 9.34 (287)	8.03 ± 7.55 (325)	9.46 ± 9.52 (326)	8.27 ± 9.82 (230)	9.03 ± 9.61 (260)	9.38 ± 10.73 (290)
T/LH ratio	3.49 ± 3.94 (2282)	4.03 ± 2.14 (244)	3.55 ± 1.88 (281)	3.56 ± 2.08 (292)	3.26 ± 1.82 (325)	3.06 ± 1.73 (346)	3.21 ± 1.98 (229)	3.36 ± 2.05 (262)	3.46 ± 2.04 (302)
BMI (kg/m^2^)	27.85 ± 4.24 (2231)	26.95 ± 3.97 (240)	27.54 ± 3.88 (268)	28.07 ± 3.92 (253)	27.18 ± 3.50 (319)	28.73 ± 4.26 (345)	27.31 ± 4.1 (252)	28.08 ± 4.16 (261)	28.62 ± 5.40 (293)
WC (cm)	99.8 ± 11.5 (2253)	97.7 ± 12.1 (242)	98.7 ± 10.6 (280)	98.9 ± 10.2 (249)	97.0 ± 9.5 (323)	102.5 ± 11.6 (341)	100.4 ± 11.7 (267)	100.8 ± 10.3 (261)	101.5 ± 14.5 (290)
Smoking (%)	17.6 (2288)	14.7 (320)	6.9 (274)	20.1 (263)	21.2 (320)	15.1 (312)	15.5 (258)	25.0 (256)	21.8 (285)

*Note*: Data are expressed as the means ± SD (number of subjects).

Abbreviations: BMI, body mass index; cFT, calculated free testosterone; FSH, follicle‐stimulating hormone; INSL3, insulin‐like peptide 3; LH, luteinizing hormone; SHBG, sex hormone‐binding globulin; T, testosterone; WC, waist circumference.

### 2.2 Questionnaires to evaluate smoking, alcohol, etc

Smoking habits were obtained by postal questionnaires, which recorded whether the participant was a current, past or non‐smoker. For this analysis, current smokers were compared with pooled past smokers and non‐smokers. Alcohol consumption was provided in terms of approximately weekly intake, whereby for multivariate analysis, 0–1 alcoholic drinks per week were allocated “low” (0), 2–4 drinks per week “moderate” (1), and >4 drinks per week “high” (2).

### 2.3 Hormone assays

Blood samples were collected before 10.00 am, and separated sera were stored at ‐80°C. Total T was measured by gas chromatography–mass spectrometry (MS)^18^ in the same laboratory for all samples. The coefficient of variation (COV) was less than 3.5% within and between runs and the detection limit was 0.17 nmol/L. LH, follicle‐stimulating hormone (FSH), and sex hormone‐binding globulin (SHBG) were all measured by the Modular E170 platform electrochemiluminescence immunoassay (Roche Diagnostics, Mannheim, Germany). The within‐ and between‐assay COVs for LH were 1.88% and 3.01%, respectively (detection limit 0.10 U/L), for FSH were 0.9% and 1.9% (detection limit 0.10 U/L), and for SHBG were 1.70% and 3.18% (detection limit 0.35 nmol/L), respectively.^19^ FT was estimated by calculation (cFT) from total T and SHBG using the Vermeulen formula.^20^ INSL3 was measured using an established and well‐validated, sensitive, and specific time‐resolved fluorescent immunoassay.^7^ The inter‐ and intra‐COVs were <8% and <3%, respectively, and the limit of detection was 20 pg/ml. In direct comparison with a new liquid chromatography–MS/MS procedure, this assay yielded almost identical values.^21^

### 2.4 Statistics

Descriptive statistics for the phase 2 subjects analyzed here are given in Table 1. Since the hormonal parameters INSL3, T, cFT, SHBG, LH, FSH, and the T/LH ratio were significantly skewed, these data were all transformed to their natural logarithm for comparison and correlation analysis, with results being back‐transformed for presentation. Since circulating INSL3 concentration, like some other parameters, appeared to decline significantly with age, these data were also normalized to a standard individual of 65 years by interpolation from the regressions of the parameter against age (expressed as the mean and 95% confidence intervals, CI). To identify potential relationships, lifestyle, hormonal, and anthropometric data were first compared by simple correlation analysis using Pearson's *R*, with INSL3, T, and cFT (Table 2), subsequently also adjusting for age and/or center. For multiple regression analysis, because of collinearity T, but not cFT, and BMI, but not WC, were included. Data were entered stepwise backward, although stepwise forward yielded identical sets of predictors. All statistics were carried out using the SPSS package (version 27; IBM SPSS Statistics for Windows; IBM Corporation, Armonk, NY, USA) and/or GraphPad Prism (version 8.2; GraphPad Software, La Jolla, CA, USA).

**TABLE 2:** Correlation analysis comparing insulin‐like peptide 3 (INSL3), total testosterone (T), and calculated free testosterone (cFT) against basic hormonal, anthropometric, and lifestyle parameters

	INSL3			T			cFT		
	*R*	*p‐*Value	df	*R*	*p*‐Value	df	*R*	*p‐*Value	df
Age	−0.358	<0.001	2280	−0.096	<0.001	2280	−0.333	<0.001	2245
INSL3				0.306	<0.001	2280	0.441	<0.001	2245
T	0.306	<0.001	2280				0.841	<0.001	2245
cFT	0.441	<0.001	2245	0.841	<0.001	2245			
SHBG	−0.165	<0.001	2245	0.479	<0.001	2245	−0.058	0.006	2245
LH	−0.224	<0.001	2244	0.152	<0.001	2244	*‐0.026*	*0.211*	*2244*
FSH	−0.252	<0.001	2242	−0.076	<0.001	2242	−0.249	<0.001	2242
T/LH ratio	0.391	<0.001	2244	0.540	<0.001	2244	0.582	<0.001	2244
BMI	−0.091	<0.001	2191	−0.334	<0.001	2191	−0.203	<0.001	2191
WC	−0.134	<0.001	2210	−0.336	<0.001	2210	−0.238	<0.001	2175
Smoking	*‐0.027*	*0.200*	*2193*	0.096	<0.001	2193	0.069	<0.001	2159
Alcohol	0.067	0.003	1981	*‐0.003*	*0.888*	*1981*	*0.044*	*0.052*	*1980*

*Note*: T, cFT, sex hormone‐binding globulin (SHBG), luteinizing hormone (LH), and follicle‐stimulating hormone (FSH) were all log_N_ transformed. Pearson's *R* represents the correlation coefficient, with degrees of freedom (df), and significance (*p*‐value). Italic values indicate a lack of significant correlation.

Abbreviations: BMI, body mass index; WC, waist circumference.

## 3 RESULTS

### 3.1 Characteristics of the EMAS cohort

The EMAS cohort at baseline has been described in detail in several previous publications.^17^, ^19^ Similarly, the follow‐up longitudinal phase of the study at an average of 4.3 years after initial sampling has also been described.^16^, ^22^ For the present study, 2283 serum samples were available for INSL3 measurement from all eight centers (approximately 250–300 per center) for phase 2, but only 1252 serum samples from four centers (approximately 300 per center) for phase 1. The descriptive statistics of the available samples (phase 2, Table 1; phase 1, not shown) did not differ significantly from those of the original cohort, as described previously.^16^, ^22^ Only the phase 2 samples were used for the detailed cross‐sectional analysis, whereas all paired samples from four centers were used for the longitudinal analysis.

### 3.2 Between‐center differences in INSL3

**FIGURE 1:**
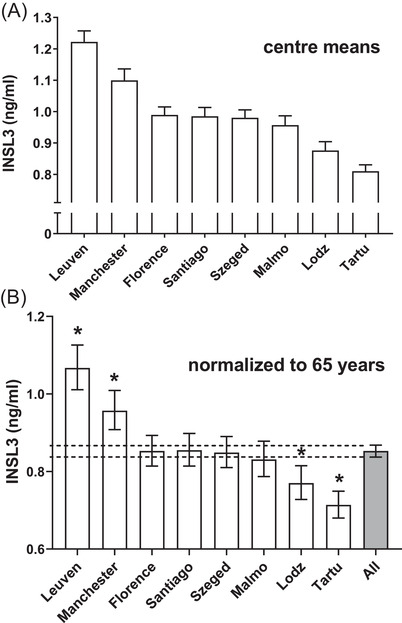
Mean insulin‐like peptide 3 (INSL3) concentration per center. (A) Simple means ± SEM. (B) Adjusted means and 95% confidence intervals (CIs) calculated by interpolation from individual INSL3 × age regressions for age 65 years. The INSL3 concentration was transformed to log_N_ for regression and back‐transformed for presentation. Asterisk (*) indicates a significant difference from the overall mean and 95% CIs (dashed lines) (*p* < 0.05)

The EMAS cohort comprises men from eight different countries, representing the northern, eastern, southern, and western regions of Europe, in approximately equal numbers. While between‐center differences for most parameters were minimal (Table 1), there were significant differences between centers for INSL3 for phase 2 (Figure 1A). Similar differences were also seen for the four centers for which data are available for phase 1 (not shown). In phase 2, Leuven registered the highest mean INSL3 (1.21 ng/ml), followed by Manchester (1.08 ng/ml), with Tartu and Lodz registering the lowest mean INSL3 values (0.81 and 0.87 ng/ml, respectively). These differences were significant (*p* < 0.05). To adjust for age, the INSL3 concentration (mean and 95% CI) at a given age of 65 years (close to the actual mean age of the cohort; 63 ± 10 years) was calculated by interpolation from the center‐specific regressions of INSL3 against age (Figure 1B). Because mean age was similar between centers, these age‐adjusted results are essentially comparable to the simple means (Figure 1A).

Data were combined for subsequent analyses, with center differences being taken into account when adjusting parameters (see later).

### 3.3 Effect of age on INSL3 and other reproductive hormones at baseline and follow‐up

**FIGURE 2:**
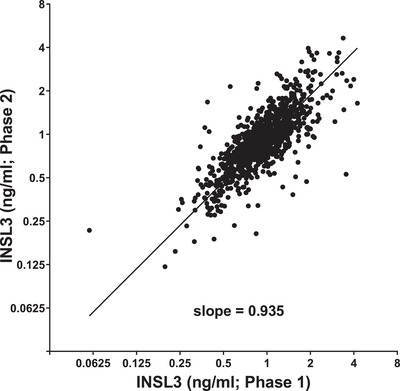
Linear regression of insulin‐like peptide 3 (INSL3) concentration from phases 1 and 2 within subjects (mean 4.3 years apart) from four centers (Florence, Manchester, Santiago, Szeged) (*n* = 961; slope = 0.935, Pearson's *R* = 0.73, *p* < 0.0001)

The regression of the phases 1 and 2 data for the four centers (*n* = 961) for which data from both phases were available shows that the within‐individual change in INSL3 concentration with age is minimal (Figure 2; Pearson's *R* = 0.73). The slope (0.935) implies a mean 6.5% reduction in INSL3 over 4.3 years, or 15.1% per decade.

**FIGURE 3:**
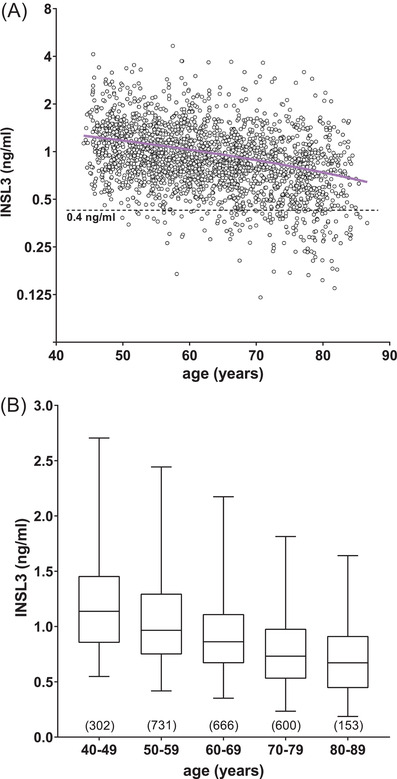
Scatterplot (A) of circulating insulin‐like peptide 3 (INSL3) concentration against age for all phase 2 subjects within the European Male Aging Study (EMAS) cohort. The solid regression line (*n* = 2325; slope = ‐0.015, *R* ^2^ = 0.09, *p* < 0.0001) is shown in magenta; the dashed line indicates a concentration of 0.4 ng/ml, equivalent to the 5% confidence interval (CI) for the entire cohort, and as a borderline to distinguish potentially hypogonadal subjects. Box and whisker plot (B) of the same data expressed as medians with 2.5%, 25%, 75%, and 97.5% CIs (*n* values in parentheses)

In the cross‐sectional analysis of phase 2 (Figure 3), INSL3 declined at 14.5% per decade. Comparing the phase 2 slope with that for phase 1 (12.5%; not shown) indicated a small, non‐significant difference largely because of the relatively higher INSL3 values in the younger age groups in phase 2 than in the similarly aged subjects from phase 1. There was less difference in the mean INSL3 between the phases for the older decades. In Figure 3A, a horizontal dashed line is drawn at 0.4 ng/ml (fifth percentile for the whole cohort) to emphasize the large increase in potentially hypogonadal subjects between ages 40 and 85 years. Figure 3B indicates the means, quartiles, and 95% CI for each decade, implying changing reference ranges depending upon the age of the subject.

Comparing cross‐sectional data from phase 2 for T, cFT, LH, and the T/LH ratio using simple regression analysis against age, T declines much more gradually (1.9% per decade) than INSL3 (Figure S1). cFT also declines significantly at 10.8% per decade. In contrast, LH increases markedly with age at an average of 23.6% per decade, with the result that the T/LH ratio consequently declines significantly at 14.3% per decade. SHBG also increased at 15.5% per decade, and FSH increased at 33.4% per decade. All regressions were highly significant (Figure S1).

### 3.4 Relationships between INSL3, total T, cFT, and other hormonal, anthropometric, and lifestyle parameters

Simple unadjusted correlation analysis (Table 2) indicated that INSL3 was significantly positively correlated with total T, cFT, the T/LH ratio, and alcohol intake. It was negatively correlated with age, SHBG, LH, FSH, BMI, and WC. The results for T show that T is positively correlated with SHBG, LH, and smoking, although not alcohol (Table 2). The correlation matrix for cFT is essentially similar to that for T, although it correlates negatively with SHBG. When subsequently adjusted for subject age ± center (not shown), partial correlation analysis shows that correlations are still essentially similar to those in Table 2, although INSL3 is now also significantly negatively correlated with smoking (*R* = ‐0.110, *p* < 0.001, *n* = 1874), as well as to alcohol consumption (*R* = 0.067, *p* = 0.004, *n* = 1874).

Table 3 presents the results of using stepwise multiple regression analysis to model INSL3 and T as dependent variables in the EMAS cohort. The standardized beta coefficients (Table 3; Std. *β*) indicate that for INSL3, where the *R* ^2^ shows that 30% of the variance is accounted for by the model, T contributes most, followed by SHBG, LH (negatively), age, smoking (negatively), and FSH (positively). For T, where the *R* ^2^ indicates that almost 50% of variance is accounted for by the model, SHBG contributes most, followed by LH (positively), FSH (negatively), INSL3, BMI, and age. The sequential, stepwise analysis means that only those parameters that significantly contribute to the variance of the dependent variable (INSL3 or T) are included in the models, with non‐significant parameters excluded (Table 3; excl.). The size of the standardized beta coefficient reflects the relative importance of those parameters, noting that gonadotropins appear to be more important for T than for INSL3. Modeling of cFT is essentially similar to that for T (not shown).

**TABLE 3:** Multiple regression analysis modeling insulin‐like peptide 3 (INSL3) and total testosterone (T) using the hormonal, anthropometric, and lifestyle parameters, as indicated

	INSL3			T		
	Std. *β*	*t*	*p*‐Value	Std. *β*	*t*	*p*‐Value
Age	−0.204	−8.62	<0.001	−0.159	−8.09	<0.001
INSL3				0.320	17.71	<0.001
T	0.443	17.70	<0.001			
SHBG	−0.259	−9.89	<0.001	0.539	28.16	<0.001
LH	−0.219	−7.56	<0.001	0.344	14.51	<0.001
FSH	0.068	2.31	0.021	−0.319	−13.19	<0.001
Smoking	−0.092	−4.59	<0.001	excl.	excl.	excl.
Alcohol	0.047	2.43	0.015	excl.	excl.	excl.
BMI	−0.044	−2.09	0.037	−0.111	−6.362	<0.001

*Note*: Waist circumference was not included in the analysis as being colinear with body mass index (BMI). Std. *β* is the standardized beta coefficient for the model with its *t*‐value and significance (*p*‐value). “excl.” indicates a parameter excluded through lack of statistical significance in the final optimal model.

Abbreviations: FSH, follicle‐stimulating hormone; LH, luteinizing hormone; SHBG, sex hormone‐binding globulin.

### 3.5 Effect of weight gain or loss for individual men between phases 1 and 2

**FIGURE 4:**
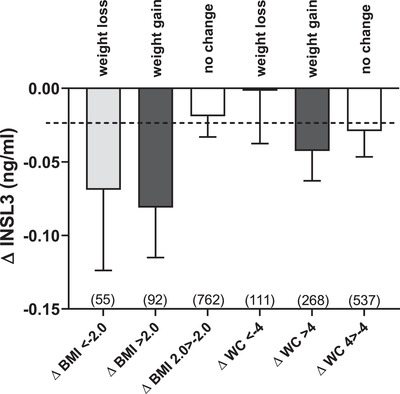
Differences in insulin‐like peptide 3 (INSL3) concentration (ΔINSL3, ng/ml; mean ± SEM) within individuals between phases 1 and 2 for men who either lost weight markedly (body mass index [ΔBMI] ≤ ‐2 kg/m^2^ or waist circumference [ΔWC] ≤ ‐4 cm) or gained weight (ΔBMI ≥ 2 kg/m^2^ or ΔWC ≥ 4 cm), compared to subjects indicating little weight change (ΔBMI ‐2 > 2 kg/m^2^ or ΔWC ‐4 > 4 cm) during the 4.3 years between phases. The dashed line indicates the change in average INSL3 concentration because of the age‐dependent decline. The numbers of subjects in each category are indicated in parentheses

For those individuals who indicated substantial changes (weight loss or weight gain) in either BMI (±2 units) or WC (±4 cm) over the 4.3 years (average) between the two phases of the study, this had no significant impact on circulating INSL3 levels (Figure 4). The generally negative effect on INSL3, irrespective of weight gain or loss, can largely be attributed to the age‐linked association alluded to above (Figure 2), whereby INSL3 is reduced on average by 15.1% per decade; this is represented by the dashed line in Figure 4. Similar analyses were carried out for T, cFT, and SHBG (Figure S2) largely confirmed the results from a previous study,^23^ whereby SHBG was significantly affected by weight loss; T showed a similar trend, although because of the reduced numbers than the previous study, this did not attain significance; cFT showed no effect.

## 4 DISCUSSION

### 4.1 Effect of age

INSL3 and T originate exclusively from the small (ca. 100–500 million) population of Leydig cells in the testes.^24^ Circulating T is dependent on the functional status of the HPG axis and on levels of steroid‐binding proteins, especially SHBG, both of which show considerable circadian and day‐to‐day variation. T production decreases in aging men,^19^, ^25^ although the extent to which this is because of a loss or dedifferentiation of Leydig cells is still largely unknown. A concomitant aging‐related increase in gonadotropins indicates primary suppression of testicular function. However, if a man gains weight with age,^19^ reduced gonadotropin‐releasing hormone pulsatility and LH secretion may ensue, indicating secondary HPG hypofunction.^25^ The adult Leydig cell population established at puberty remains relatively constant in adult life, with some^3^, ^4^ although not all^24^ studies suggesting there may be some attrition of Leydig cells in old age. While the role of pulsatile LH in stimulating both Leydig cell proliferation and differentiation during puberty is well established, the regulation of Leydig cell dynamics in adult life is less well understood and is unlikely to be fully monitored by T measurement.

The present study confirmed the cross‐sectional age‐dependent decline in INSL3, T, and cFT between ages 40 and 80 years, previously identified.^14^ In particular, the EMAS cohort confirms the ca. 14.5% per decade decline in INSL3 compared to the slower decline in circulating T (1.9% per decade) and cFT (10.8% per decade), which are compensated by the marked increase in LH (23.6% per decade) and SHBG (15.5% per decade) with increasing age (Figure S1). Because INSL3 production by Leydig cells is essentially constitutive and is a biomarker of the more mature Leydig cell,^8^ circulating INSL3 concentration can be seen as a reliable index of mature Leydig cell numbers and their differentiation status, reflecting their functional capacity. This view is supported by the similar decline of ca. 14.3% per decade in the T/LH ratio (Figure S1D), also regarded as an index of Leydig cell functional capacity,^26^, ^27^ and by the comparable decline (44% over 30 years) reported for mature Leydig cell numbers.^3^ This is the first study, however, to provide longitudinal information on the within‐individual decline in INSL3 of 15.1% per decade, which is again very similar to the calculated cross‐sectional decline in INSL3 (14.5% per decade) and the T/LH ratio (14.4% per decade). Similar reductions were recorded earlier in a cross‐sectional cohort of Australian men (INSL3, ca. 13.6% per decade; T/LH ratio, 22.7% per decade).^14^

In a recent study of a much more homogeneous cohort of military conscripts, all healthy and aged approximately 18 years, there was no significant correlation between INSL3 and the T/LH ratio.^28^ This suggests that the two parameters indeed differ, with the former a more anatomical construct and the latter more physiological, also reflecting the cells’ sensitivity to LH. This could be understood when one considers that the aged testis may become highly fibrotic with marked focal atrophy of seminiferous tubules and sclerotic vasculature hampering the access of circulating hormones to the interstitial compartment and vice versa. In contrast, the testis in young men is more homogeneous, with more effective blood perfusion. Thus, the T/LH ratio more likely reflects the ability of LH to access Leydig cells and for their products to exert feedback inhibition on the HPG axis, whereas circulating INSL3 more likely reflects the actual anatomical status.

An important finding of this study is that the longitudinal data collected on average 4.3 years apart, and with low within‐individual variance, imply that the large, ca. 10‐fold range of INSL3 values seen in younger and middle‐aged men persists into older age. An individual who has low INSL3 at age 40 years is therefore likely to have low INSL3 and potentially functional hypogonadism in older age, suggesting that INSL3 has important predictive value as a clinical parameter. Although the comparison of phases 1 and 2 values for T within individuals offers a similar correlation coefficient to that for INSL3 (*R* ^2 ^= 0.55 vs. 0.53, respectively, not shown), the cross‐sectional correlation of T with age is substantially weaker than for INSL3 (*R* = ‐0.096 vs. ‐0.358, respectively; Table 2), suggesting the marked influence of other factors to increase the variance in T over longer time periods.

### 4.2 Effect of the HPG axis

The INSL3 concentration correlates well (Table 2) with the hormones of the HPG axis, especially T and cFT, as well as with the T/LH ratio in this cohort. INSL3 differs from T (total or free) in that it correlates negatively with both FSH and LH, whereas T correlates positively with LH but negatively with FSH. This is to be understood in the context of the acute mode of regulation by LH, and not by FSH, of Leydig cell steroidogenesis but not of INSL3 production. INSL3 is higher in healthier and younger individuals, where there is less requirement for acute LH output to compensate for reduced T production. Circulating T negatively correlates with FSH because an increase in the latter is often a sign of primary testicular failure. In younger, healthier men, testicular output is high and requires lower amounts of gonadotropins for its maintenance, in part regulated by negative feedback of inhibin B from Sertoli cells^29^; the converse is true for older, less healthy, and possibly sclerotized testes. Interestingly, in the multiple regression analysis (Table 3), where we have attempted to account for all possible confounding interactions between parameters in the construction of explanatory models for INSL3 and T, FSH now becomes a positive factor in determining INSL3 levels. This was also observed for the younger cohort of Swedish military conscripts^28^ and suggests a role for Sertoli cells in mediating the regulation of INSL3, although not of T.

### 4.3 Effect of obesity and lifestyle factors

Of the lifestyle parameters measured, and as shown earlier,^15^, ^28^, ^30^ both higher BMI and WC appear to have a similar negative impact on INSL3, T, and cFT (Table 2). Although a different analysis, simply comparing the effect of obesity (BMI > 30) on the levels of INSL3, T, cFT, and SHBG for the phase 2 subjects also indicated a significant impact of obesity on INSL3, as well as on the other parameters (Figure S3); the impact is relatively smaller (‐9.4%) for the former than T (‐21.5%), cFT (‐14.5%), and SHBG (‐17.4%). For T, this has been linked partly to the ability of adipose tissue to use aromatase to convert androgens to estrogens,^31^, ^32^ although direct effects of obesogens or inflammatory cytokines on the testes are not excluded.^32^ Partly also, this may be because BMI can influence SHBG levels (Figure S2), which in turn may regulate hypothalamic feedback and hence gonadotropin production.^33^ Although the INSL3 results suggest that there may be a small impact of obesity on Leydig cell functional capacity, multiple regression analysis (Table 3) shows that the effect of BMI becomes negligible for INSL3, although not for T. Similarly, although weight loss can lead to an increase in circulating T^23^, ^32^ and consequent health improvement, the results in Figure 4 show that neither substantial weight loss nor weight gain over an average 4.3‐year period in older aged men have any long‐term impact on Leydig cell functional capacity, as represented by INSL3.

It is important to distinguish these findings from more extreme situations where for example, young hypogonadal patients have been given LH or human chorionic gonadotropin for prolonged periods of weeks or months^34^ or where subjects are recovering from contraceptive suppression of the HPG axis.^35^ Such interventions indeed lead to increased circulating INSL3. INSL3 was recently assessed in body builders using anabolic steroids to promote muscle development.^36^ Not only did INSL3 become reduced in these subjects because of loss of Leydig cell functional capacity, but some of this reduction persisted even after several months of stopping the anabolic steroids and consequent recovery of the HPG axis. The testes probably contain some less differentiated Leydig cells,^37^ which in these younger men may redifferentiate to some degree under chronic gonadotropin stimulation, although it should be noted that previous high levels of INSL3 are not fully regained.

Correlation analysis indicates that smoking acts negatively on INSL3 but positively on T (see earlier; Table 2), as shown in previous studies.^15^, ^28^ Multiple regression analysis confirms the negative impact of smoking on INSL3, although the effect on T is now negligible (Table 3). This implies different mechanisms of action for smoking in relation to the two hormones. Moderate alcohol consumption appears to have only a small effect on INSL3 but not on T (Tables 2 and 3).

### 4.4 Geographic variation in INSL3 concentration

**FIGURE 5:**
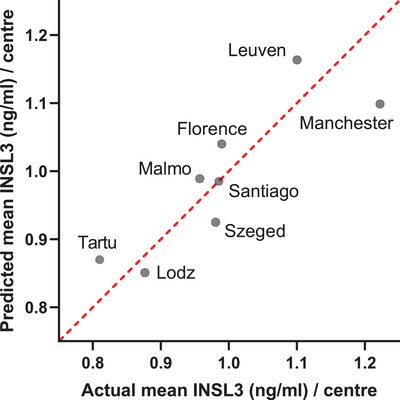
Plot of actual mean insulin‐like peptide 3 (INSL3) concentration (ng/ml) for each European Male Aging Study (EMAS) center against the center means predicted by multiple regression analysis using key lifestyle parameters (waist circumference [WC], smoking, alcohol), whereby the two former parameters contributed most to the variation ([INSL3 predicted] = 4.747 – 0.036[WC] – 0.013[smoking] + 0.007[alcohol])

Leuven and Manchester had significantly higher mean circulating INSL3 concentrations than the total cohort average, and Lodz and Tartu had significantly lower concentrations. In contrast, the T concentration indicates only small between‐center differences (Table 1). Inspection of the center descriptive statistics does not immediately suggest any major single parameter difference between the centers. However, a multiple regression analysis of the center means for INSL3 and the means for plausible lifestyle parameters (Figure 5) suggests that WC (or BMI), smoking, and alcohol consumption, could explain much (estimated explained variance based on *R* ^2^ = 73.2%), although not all, of the center‐specific variation in these older men. Other influences could include reduced general health status and genetic or environmental factors. Such geographic variation could be considered in the establishment of clinical reference ranges for INSL3. Based on the total EMAS data for all centers, INSL3 reference ranges (95% CI; Figure 3B) should vary depending on age, at least above 40 years, declining from 0.6–2.4 ng/ml (age 40–49 years) to 0.3–1.6 ng/ml (age 80–89 years). This differs somewhat from the observations of Albrethsen et al.^30^ who found little age‐dependent change within a Danish cohort of younger men (age 19–40 years, 0.9–2.7 ng/ml; age 50–60 years, 0.9–2.5 ng/ml). We also observed a similar INSL3 reference range for a cohort of 18‐year‐old Swedish men (0.4–1.9 ng/ml).^28^

## 5 CONCLUSION

Together, these findings show that in normal subjects, Leydig cell functional capacity as reflected in circulating insulin‐like peptide 3 is highly consistent within an individual over long periods and that insulin‐like peptide 3 is a powerful long‐term index of Leydig cell status.^38^ Importantly, it could be a predictor of later health and/or hypogonadism; it is a robust and easily measurable parameter with little short‐term biological variability. A limitation of this study is that its retrospective nature limited the list of possible associations contributing to insulin‐like peptide 3 variance; future studies will no doubt expand on such factors.

## CONFLICT OF INTEREST

The authors declare that they have no conflicts of interest.

## AUTHOR CONTRIBUTIONS

Ravinder Anand‐Ivell supervised the INSL3 measurement and its validation, and contributed to the conception, analysis, and writing of this study. Kee Heng was responsible for INSL3 measurement. Katie Severn advised and supported the statistical analysis. Ilpo T. Huhtaniemi and Frederick C.W. Wu were responsible for the provision and coordination of EMAS samples and data and contributed to the conception and writing of this study. Leen Antonio, Gyorgy Bartfai, Felipe F. Casanueva, Aleksander Giwercman, Mario Maggi, Terence W. O'Neill, Margus Punab, Giulia Rastrelli, Jolanta Slowikowska‐Hilczer, Jos Tournoy, and Dirk Vanderschueren helped with data collection, reviewing, commenting on and approving the final manuscript. Richard Ivell was responsible for the overall conception, analysis, and writing of the study.

## Supporting information

Supplementary material

Supplementary material
