# Supplementary material for: Association of age, hormonal, and lifestyle factors with the Leydig cell biomarker INSL3 in aging men from the European Male Aging Study cohort
Source: Andrology. 2022 Jul 11;10(7):1328–38. doi: 10.1111/andr.13220 (PMC9540576; doi:10.1111/andr.13220)
Supplement: Supplementary file 1 — Supplementary material [file ANDR-10-1328-s002.docx]

**Legends to Supplementary Figures**

***Suppl. Figure 1***

Scatterplot regressions of testosterone (A), calculated free testosterone (B), SHBG (C), the T/LH ratio (D), LH (E), and FSH (F) against age for the entire EMAS cohort, indicating the slope, Pearson R^2^, and its significance. Correlations were calculated from the log-transformed data using the non-linear function of GraphPad Prism version 8.2.

***Suppl. Figure 2***

Differences in (**A**) total testosterone (T), (**B**) calculated free testosterone (cFT), and (**C**) SHBG concentrations within individuals between phases 1 and 2, for men who have either lost weight markedly (ΔBMI < -2 kg/m^2^ or ΔWC (waist circumference) < -4 cm), or gained weight (ΔBMI > 2 kg/m^2^ or ΔWC > 4 cm), compared to subjects indicating little weight change (ΔBMI -2 > 2 kg/m^2^ or ΔWC -4 > 4 cm) during the 4.3 years between phases (means + SEM). The dashed lines indicate the change in average parameter concentration due to age alone. The numbers of subjects in each category are indicated in parentheses. * and *** indicate statistically significant differences at *p*<0.05 and *p*<0.0001 levels, respectively.

***Suppl. Figure 3***

Effect of being obese (BMI>30) or not obese (BMI<30) on the age-corrected levels of INSL3, total testosterone (T), calculated free testosterone (cFT), and SHBG. Data represent the interpolated means (95% CI) for a 65-year-old man derived from individual parameter vs age regressions. * indicates significant difference (p<0.05).
