# Supplementary figures and images for: Association of age, hormonal, and lifestyle factors with the Leydig cell biomarker INSL3 in aging men from the European Male Aging Study cohort
Source: Andrology. 2022 Jul 11;10(7):1328–38. doi: 10.1111/andr.13220 (PMC9540576; doi:10.1111/andr.13220)

# Suppl Fig 1

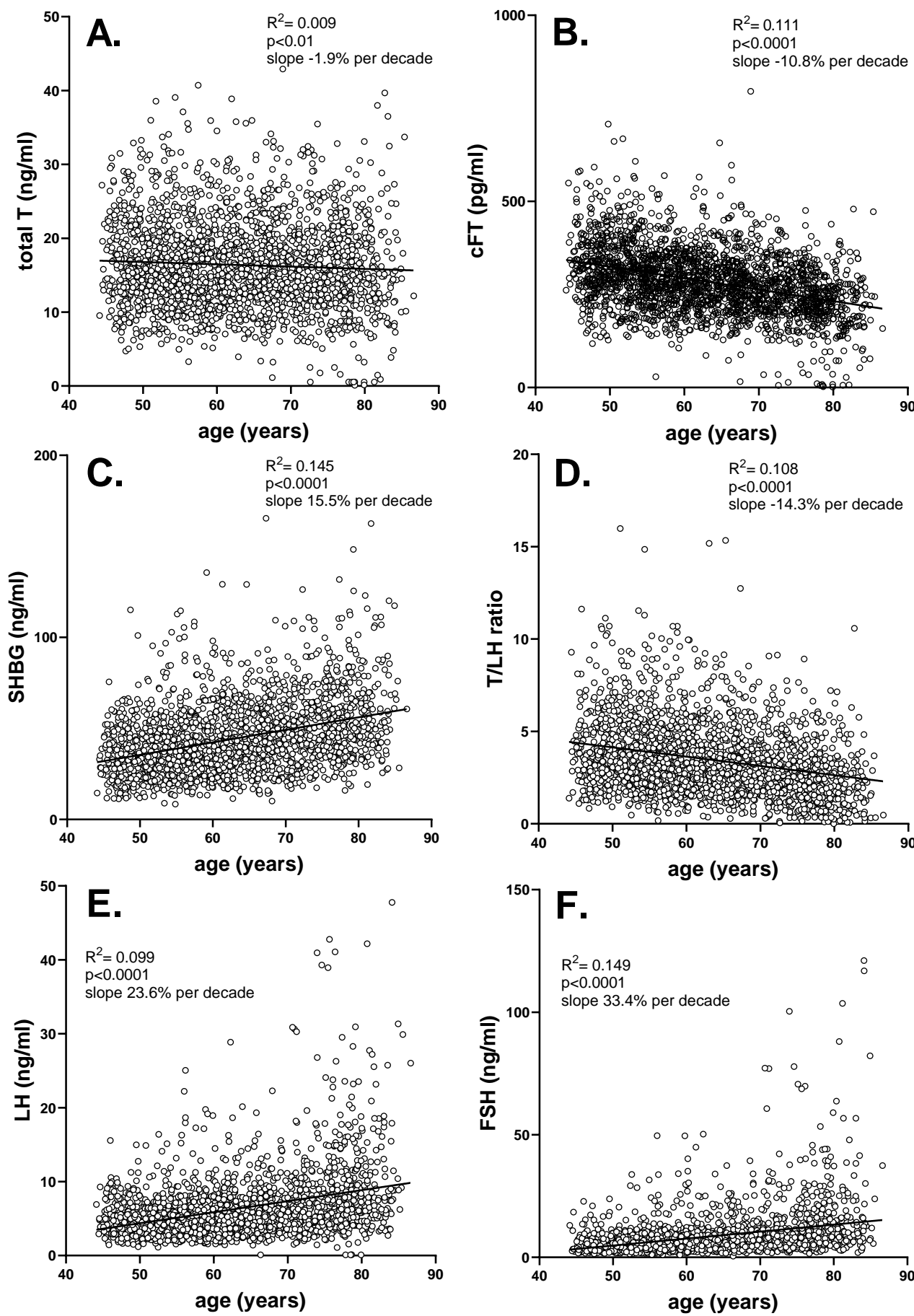

# Suppl Fig 2

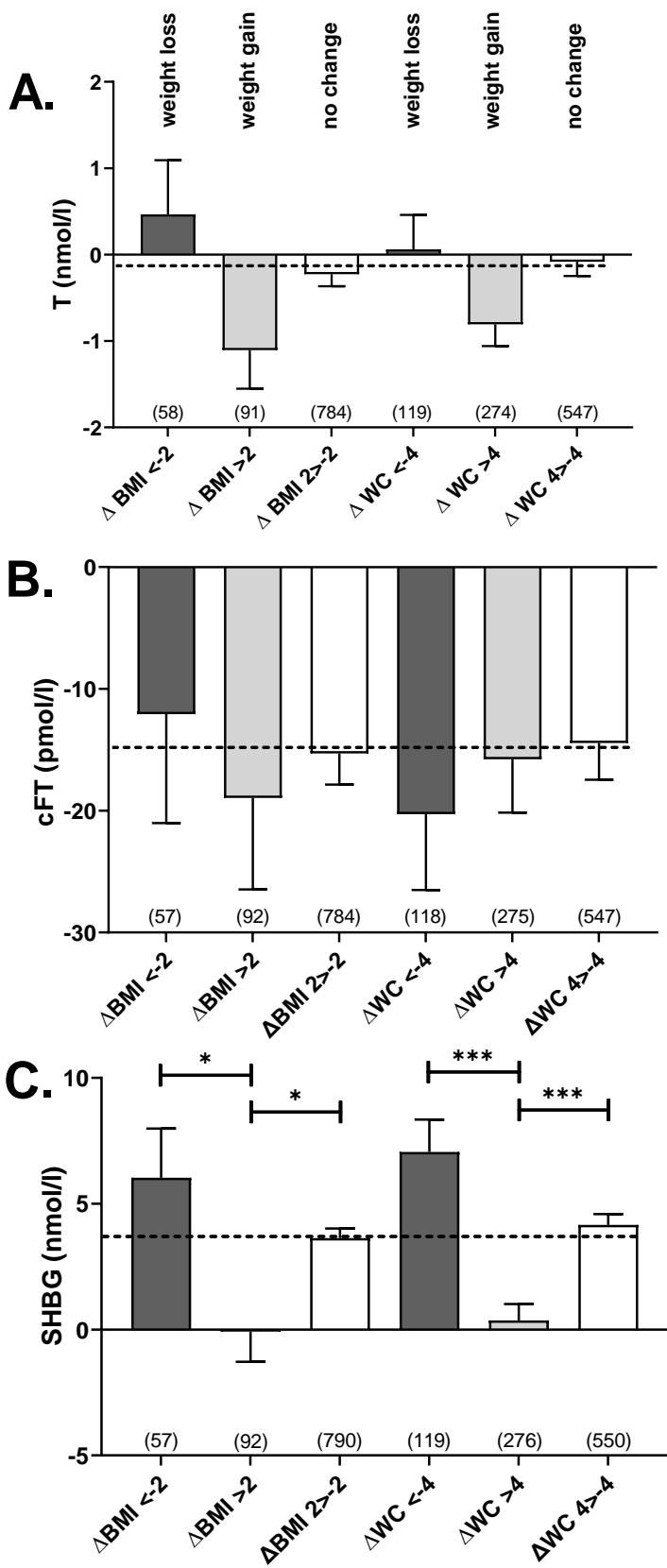

Suppl Fig 3

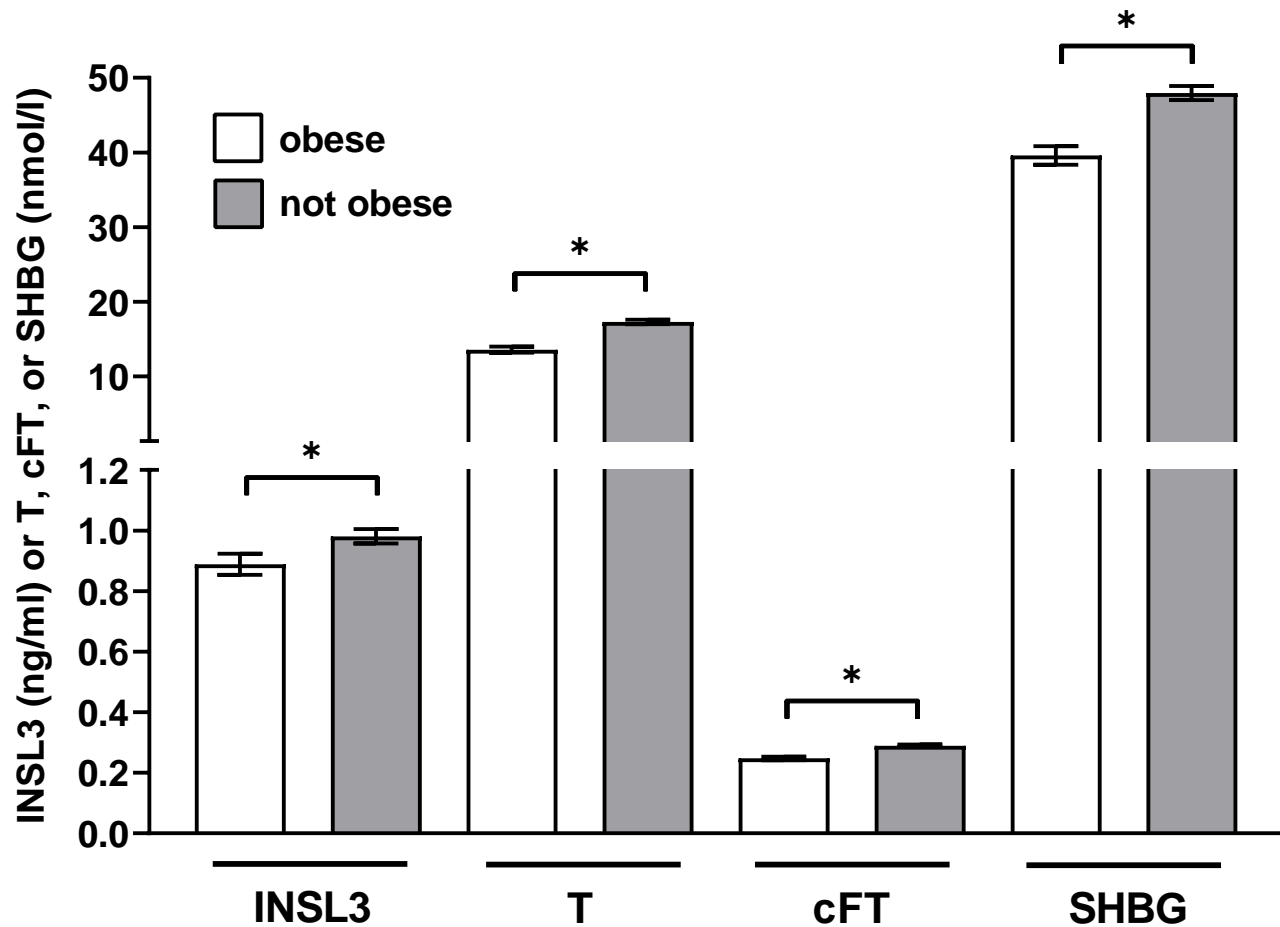

Supplement: Supplementary file 2 — Supplementary material [file ANDR-10-1328-s001.pdf]
